# Pain, Physical Activity, and Subjective Memory Complaints: Evidence from the Health and Retirement Study

**DOI:** 10.1093/geroni/igaf122.3232

**Published:** 2025-12-31

**Authors:** Chang Pu Liang, Wuyi Dong, Yan-Jhu Su

**Affiliations:** University of Massachusetts Boston, Boston, Massachusetts, United States; University of Massachusetts Boston, Boston, Massachusetts, United States; The Pennsylvania State University, University Park, Massachusetts, United States

## Abstract

This study investigates the intricate relationships between physical activity, pain, and subjective memory complaints (SMCs) among older adults using data from the 2020 Health and Retirement Study. The primary aims were to examine the association between physical activity and SMCs, evaluate the independent contribution of pain to SMCs, and explore the potential moderating role of pain in the relationship between physical activity and SMCs. Results from linear regression models indicated that the associations of pain and physical activity with SMCs were significant in unadjusted analyses but became non-significant after controlling for covariates, suggesting that self-rated health and depression may mediate these relationships. Stratified analyses further confirmed these findings, showing that physical activity did not significantly predict SMCs in either pain-stratified group. Interaction terms between physical activity and pain were not significant, indicating no moderating effect of pain on the physical activity-SMCs relationship. These results emphasize the importance of promoting physical activity and managing pain to support cognitive health in older adults, and the need for further research to elucidate underlying mechanisms and mediating roles of self-rated health and depression.

